# An ELISA-Based Alternative to Mouse Bioassays for Quantitative Evaluation of Tetanus Toxin

**DOI:** 10.3390/toxins18030133

**Published:** 2026-03-09

**Authors:** Chie Shitada, Chiyomi Sakamoto, Kohsuke Kumeda, Susumu Yamaori, Motohide Takahashi

**Affiliations:** 1 Toxin and Biologicals Research Laboratory, Kumamoto Health Science University, 325 Izumi-machi, Kita-ku, Kumamoto 861-5533, Japan; sakamoto.c01@kumamoto-hsu.ac.jp; 2 The Chemo-Sero-Therapeutic Research Institute (KAKETSUKEN), 8-7 Shinshigai, Chuou-ku, Kumamoto 860-0803, Japan; 3 KM Biologics Co., Ltd., 1-6-1 Okubo, Kita-ku, Kumamoto 860-8568, Japan; kumeda@kmbiologics.com (K.K.); yamaori-su@kmbiologics.com (S.Y.)

**Keywords:** tetanus toxin, *Clostridium tetani*, 3Rs, ELISA, immunochromatography

## Abstract

Tetanus toxin evaluation has traditionally relied on mouse LD_50_ bioassays, which require extensive animal use and time, necessitating development of alternative methods in accordance with 3R principles (Replacement, Reduction, and Refinement). We developed and validated a sandwich enzyme-linked immunosorbent assay (ELISA) as an alternative to animal testing for evaluating tetanus toxin biological activity using 18 environmental and clinical isolates of *Clostridium tetani*, complemented by an immunochromatographic (IC) assay for rapid screening. The ELISA demonstrated excellent analytical performance with a lower limit of quantification of 2.4 ng/mL (equivalent to 85.4 LD_50_/mL), favorable linearity (R^2^ = 0.999), precision (CV < 1.7–8.2%), and specificity (<1% cross-reactivity with *C. septicum*, *C. novyi*, and *C. perfringens*). Correlation analysis between ELISA relative potency and observed minimum lethal dose values revealed a robust positive correlation (r = 0.974). Both parallel line assay and single-point quantification methods showed strong correlations with mouse bioactivity measurements (r = 0.998). The IC assay successfully detected all isolates within 15 min. The measurement range of 2.4–45.6 ng/mL effectively covered diverse toxin-production capabilities spanning a 600-fold concentration range. This validated ELISA and IC assay combination provides a reliable, rapid alternative to animal experimentation for tetanus toxin evaluation.

## 1. Introduction

Tetanus is a disease caused by a potent neurotoxin produced by *Clostridium tetani* and remains a significant public health concern [1]. The global burden of tetanus shows significant regional variations, with high incidence rates particularly in Africa, South Asia, and Southeast Asia, which are often associated with an agricultural lifestyle and inadequate vaccination coverage [2]. The evaluation of tetanus toxin activity is crucial for vaccine quality control, assessing clinical isolates, and evaluating environmental risks. Although conventional bioassays using mice provide definitive toxin identification and quantitative evaluation through LD_50_ measurements, these methods require a large number of experimental animals and extensive time periods. From an animal welfare perspective, the development of alternative approaches based on the 3R principles (Replacement, Reduction, and Refinement) is necessary worldwide [3].

*C. tetani* is widely distributed in the environment and survives for extended periods through spore formation [4]. *C. tetani* spores exhibit remarkable environmental persistence, remain viable in soil for many years, and demonstrate resistance to extreme temperatures, desiccation, and most disinfectants [5]. In clinical settings, evaluating the toxin-production ability of a strain provides critical information for treatment decisions and prognosis [6]. Rapid toxin evaluation is crucial for assessing the risk of infection during disasters and agricultural work [1]. Agricultural workers are uniquely vulnerable to tetanus due to their frequent exposure to soil and organic matter laden with *C. tetani* spores. Activities such as plowing and handling livestock significantly amplify this risk [7]. During large-scale natural disasters, compounding factors such as the type of injury, lack of medical services, and delayed treatment, significantly increase the incidence and risk [8]. However, current evaluation methods depend on animal testing, which presents challenges in terms of speed and efficiency.

Our previous genome analysis of 151 *C. tetani* strains isolated from soil in Kumamoto Prefecture confirmed three major phylogenetic groups, with clades 1–3 strains showing significantly higher toxin-production capabilities [9].

Enzyme-linked immunosorbent assays (ELISA) have high specificity and sensitivity for immunological measurements [10]. Amplified ELISA techniques, utilizing signal enhancement methods, have demonstrated improved sensitivity for bacterial toxin detection, approaching the sensitivity of traditional bioassays [11]. Although ELISA-based methods for tetanus antibody detection [12,13] and toxoid antigen detection in vaccines have been reported [14,15], studies on the use of ELISA as an alternative to animal experimentation for tetanus toxin antigen detection in clinical and environmental applications are limited. Detailed correlation analyses with mouse bioactivity assays are rare [16].

In this study, we developed and validated an ELISA-based system as a reliable alternative to animal experimentation for evaluating tetanus toxins, using environmental and clinical *C. tetani* isolates. We also developed a complementary immunochromatographic (IC) assay for rapid screening. This breakthrough addresses the urgent need for implementing the 3R principles in both clinical diagnosis and environmental monitoring, enabling rapid toxin evaluation for assessing infection risk during disasters and agricultural work. The dual-format approach represents a significant advancement in microbial toxin evaluation methodology while promoting animal welfare in scientific research.

Current diagnostic approaches for tetanus rely primarily on clinical recognition of characteristic symptoms, as direct pathogen isolation and identification from wound sites is often unsuccessful due to the anaerobic nature and sporadic distribution of *C. tetani* [1]. Clinical diagnosis is further complicated by the variable incubation period (3–21 days) and the fact that symptoms may not appear until significant neurological damage has occurred. Traditional laboratory confirmation through mouse bioassays, while definitive, requires several days and extensive animal resources, making it impractical for routine clinical decision-making where immediate treatment initiation is critical for patient outcomes.

Several alternative approaches have been developed for tetanus toxin and antitoxin evaluation, primarily in the context of vaccine quality control. These include TOBI (Test for Potency in Biological Products), cell-based assays (including Vero cell-based assays), and BINACLE (BINding And CLEavage assay) techniques [17,18,19]. Recent developments have demonstrated the potential of ELISA-based methods for tetanus toxoid potency testing as alternatives to animal challenge tests [20]. However, these methods are specifically optimized for vaccine standardization and quality control applications, often requiring specialized facilities, standardized reference preparations, and extended incubation periods. Their application to environmental isolates or clinical specimens presents significant challenges due to matrix interference, variable toxin concentrations, and the need for rapid results in clinical settings.

The development of immunoassay-based approaches offers several advantages for clinical and environmental applications. Quantitative assessment is essential for risk stratification, enabling differentiation between high-risk and low-risk isolates that require different clinical management approaches. Environmental monitoring applications require rapid screening capabilities to assess contamination levels during disaster response or agricultural exposure incidents. Furthermore, the variable toxin-production capabilities among *C. tetani* strains (as demonstrated in our previous phylogenetic analysis [9]) necessitates methods capable of detecting and quantifying toxin across a wide dynamic range.

The integration of both quantitative (ELISA) and qualitative rapid testing (IC) addresses complementary needs: immediate risk assessment in field settings through IC screening, followed by precise quantification through ELISA when detailed dose–response information is required for clinical or environmental risk management decisions. This dual approach represents a practical solution for implementing 3R principles while maintaining the analytical capability required for diverse tetanus toxin evaluation applications.

Previous ELISA studies for tetanus toxoid applications have demonstrated strong correlations with biological activity, with correlation coefficients reaching 0.931 between serological responses and challenge test results [20]. However, these studies primarily focused on vaccine standardization rather than clinical or environmental applications. The present study addresses this gap by developing methods specifically optimized for diverse sample matrices and rapid clinical decision-making.

While the IC assay provides rapid qualitative results, it has inherent limitations, including its semi-quantitative nature and potential for subjective interpretation of signal intensity. Future developments could incorporate fluorescent labels or digital signal quantification to enhance objectivity and enable semi-quantitative assessment.

Despite recent advances in alternative testing methods [21], no validated system exists for rapid, quantitative tetanus toxin evaluation in clinical and environmental contexts.

## 2. Results

### 2.1. ELISA Validation and Performance

**Figure 1:**
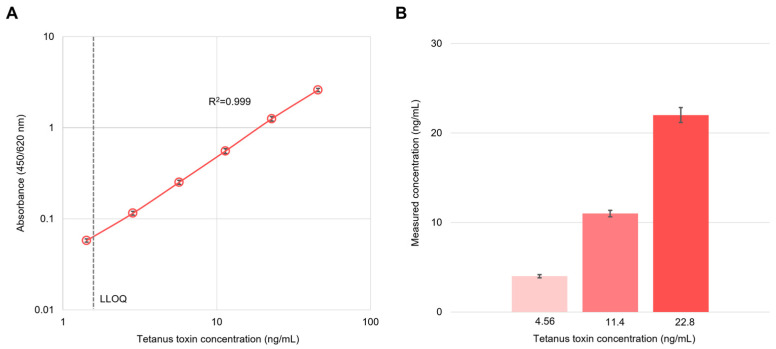
Linearity and Precision Validation of the Assay. (**A**) Calibration curve demonstrating excellent linearity (R^2^ = 0.999). For each concentration, six measurements were performed per day over three independent days (*n* = 6 per day). The daily mean was calculated, and the overall mean was derived from the three daily means (*n* = 3 days), representing inter-day variability. The lower limit of quantification (LLOQ) was determined to be 2.4 ng/mL equivalent. (**B**) Three concentration levels (nominal: 4.56, 11.4, and 22.8 ng/mL) were evaluated. Each concentration was measured eight times per day over three independent days (*n* = 8 per day; total *n* = 24). The measured mean concentrations were 4.4, 11.0, and 22.1 ng/mL, with coefficients of variation (CV) of 3.7%, 3.3%, and 3.8%, respectively. Error bars represent ±standard deviation calculated from three daily means (**A**) and from all 24 measurements per concentration (**B**).

Tetanus toxin reference standard (TT-001) demonstrated excellent analytical performance, with a precision of 1.7–8.2% (CV), excellent linearity (R^2^ = 0.999), and results accuracy to within ±15% of the theoretical values (Figure 1A). The lower limit of quantification (LOQ) was defined as the lowest concentration that could be measured with acceptable accuracy (±20%) and precision (CV ≤ 20%). The LOQ was initially calculated as the concentration corresponding to the mean blank absorbance plus 10 times the standard deviation of blank measurements, using sample diluent as the blank reference sample (*n* = 6). However, since the calculated absorbance values (0.024, 0.037) were below the reliable detection threshold (absorbance ≥ 0.1), the LOQ was established as the concentration corresponding to an absorbance of 0.1, determined to be 2.4 ng/mL. The assay measurement range was defined as 2.4–45.6 ng/mL (Table 1). Intermediate precision evaluation at three concentration levels (4.56, 11.4, and 22.8 ng/mL) showed measured mean concentrations of 4.4, 11.0, and 22.1 ng/mL, with coefficients of variation of 3.7%, 3.3%, and 3.8%, respectively (*n* = 24) (Figure 1B).

**Figure 2:**
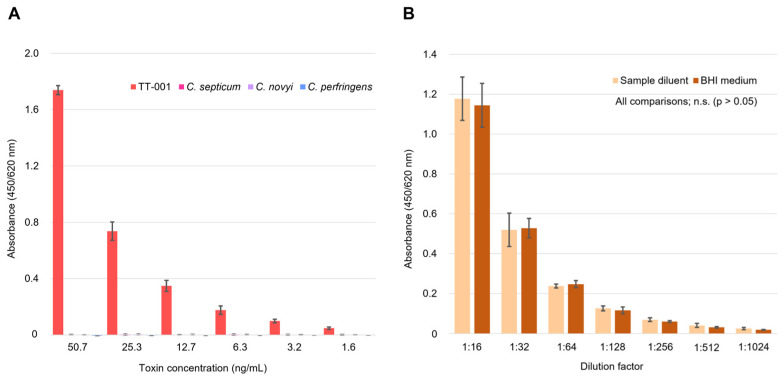
ELISA Validation Parameters. (**A**) Cross-reactivity assessment showing absorbance values of TT-001 and other Clostridium toxins (*C. septicum*, *C. novyi*, and *C. perfringens*) across the calibration range. All heterologous toxins showed < 1% cross-reactivity, confirming excellent specificity (*n* = 3). (**B**) Matrix interference evaluation by parallel line assay showing no significant differences between the sample diluent and the BHI medium across all dilution factors. Culture supernatants were initially diluted to fall within the calibration range, followed by serial dilutions for comparison with the matrix. Error bars represent ±standard deviation calculated from three independent measurements (*n* = 3). Statistical analysis showed no significant differences (*p* > 0.05, unpaired *t*-test).

Cross-reactivity assessment using heterologous Clostridium toxins demonstrated excellent specificity, with all the tested toxins (*C. septicum*, *C. novyi*, and *C. perfringens*) showing cross-reactivity values below 1% across the entire calibration range (1.6–50.7 ng/mL equivalent concentrations) (Figure 2A). Specifically, the cross-reactivity values were 0.20% for *C. septicum*, 0.18% for *C. novyi*, and 0.00% for *C. perfringens*, all meeting the acceptance criterion of <5%. Matrix interference evaluation demonstrated no significant differences between the sample diluent and the BHI medium at any dilution level (*p* > 0.05) (Figure 2B).

### 2.2. Initial Isolate Screening and MLD Prediction

The parallel line assay of 10 initial isolates (9 environmental + 1 clinical) yielded relative potency values ranging from 0.05 to 5.49 (a 110-fold difference) when compared to TT-001, using a calibration range of 85.4–1410 LD_50_/mL as the reference standard. Mouse minimum lethal dose (MLD) evaluation confirmed the predicted toxin-production capabilities, with MLD values ranging from 400 to 140,000 (350-fold difference) across all 10 isolates (Table 2). Correlation analysis between the ELISA relative potency values and observed MLD values revealed a robust positive correlation after logarithmic transformation (r = 0.981, *p* < 0.01, R^2^ = 0.963), validating the ELISA-based prediction approach.

### 2.3. Representative Strain Selection and LD_50_ Analysis

Based on the MLD evaluation results, three representative strains with distinct toxin-production levels were selected for detailed LD_50_ analysis: KHSU-134328-113 (high producer), KHSU-144313-037 (moderate producer), and KHSU-144303-003 (low producer). LD_50_ measurements of representative strains are shown in Table 2.

### 2.4. ELISA-Bioassay Correlation Analysis

**Figure 3:**
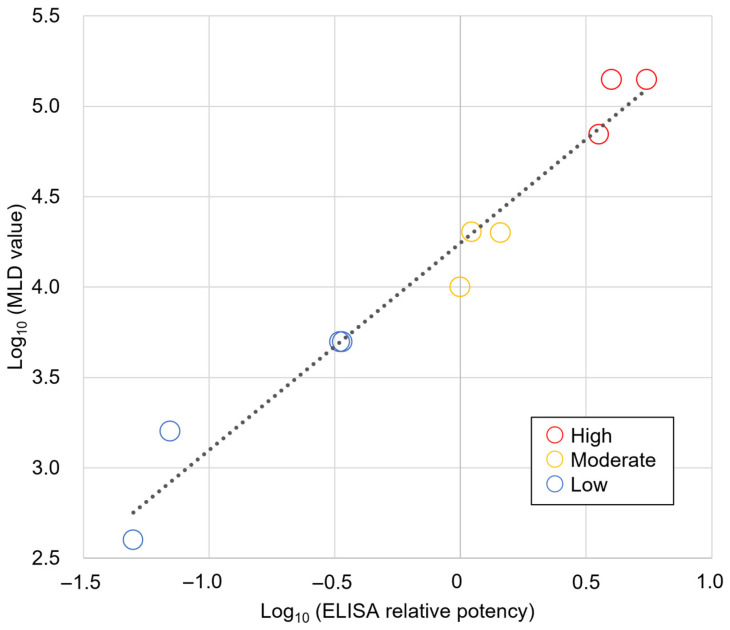
Correlation Between ELISA Relative Potency and Mouse MLD Values for *Clostridium tetani* Isolates. Correlation analysis between ELISA relative potency values and mouse MLD values for 10 initial isolates (9 environmental + 1 clinical) after logarithmic transformation showed r = 0.981, *p* < 0.01, R^2^ = 0.963. The dotted line represents the linear regression line. Color coding represents toxin-production levels: red (high), yellow (moderate), and blue (low).

The LD_50_ of TT-001 was calculated as 6.5 × 10^7^ LD_50_/mL using the probit method (Table 3). Correlation analysis between ELISA relative potency values and mouse MLD values for the 10 initial isolates showed a robust positive correlation after logarithmic transformation (r = 0.981, *p* < 0.01, R^2^ = 0.963) across a 350-fold concentration range (Figure 3).

To validate the ELISA method for LD_50_ evaluation, all 10 initial isolates were subsequently quantified using both the single-point four-parameter logistic (4PL) method and the parallel line assay with TT-001 as the reference standard (Table 4). The parallel line assay demonstrated excellent dose–response relationships for all strains, with valid parallelism and relative potencies ranging from 0.025 to 1.540. The 4PL method quantification yielded LD_50_-equivalent concentrations ranging from 1963 to 153,219 LD_50_/mL for the environmental isolates. Correlation analysis between LD_50_ values calculated by the parallel line assay and the single-point method for the 10 environmental isolates showed an excellent correlation (r = 0.998, R^2^ = 0.997, *p* < 0.001).

**Figure 4:**
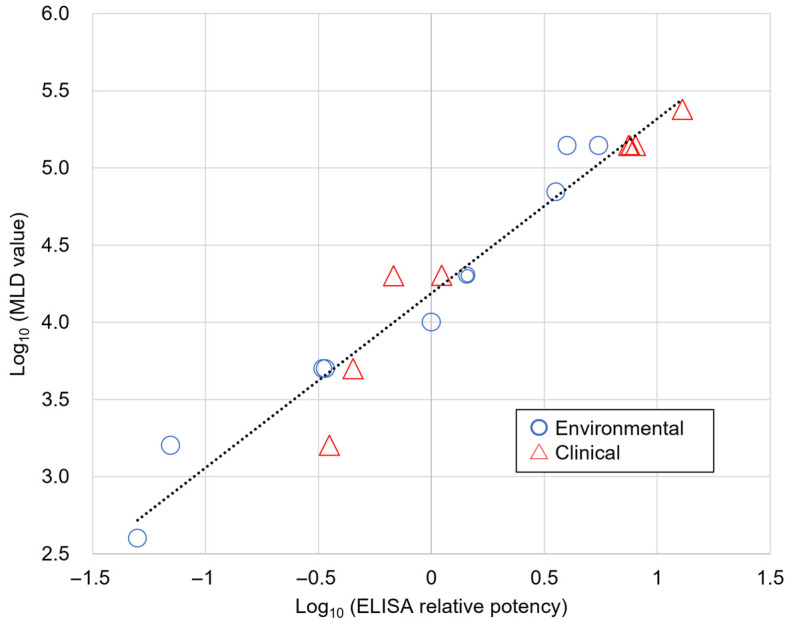
Correlation Between ELISA Relative Potency and Mouse MLD Values for *Clostridium tetani* Isolates. Correlation analysis between ELISA relative potency values and mouse MLD values for all 18 *C. tetani* isolates (9 environmental + 9 clinical) after logarithmic transformation (r = 0.974, *p* < 0.01, R^2^ = 0.948). The dotted line represents the linear regression line. Color coding distinguishes isolate sources: blue circles (environmental isolates), red triangles (clinical isolates). MLD values range from 400 to 240,000 (600-fold difference).

Based on this validation, the established ELISA methodology was applied to evaluate the 8 clinical isolates using both quantification approaches (Table 5). Comprehensive analysis combining all 18 isolates (10 initial + 8 clinical) revealed an extended range of toxin-production diversity, with ELISA relative potency values ranging from 0.025 to 12.91 (516-fold difference) and MLD values ranging from 400 to 240,000 (600-fold difference). Correlation analysis of the complete dataset showed a positive correlation between ELISA relative potency values and observed MLD values after logarithmic transformation (r = 0.974, *p* < 0.01, R^2^ = 0.948) (Figure 4).

### 2.5. Immunochromatographic Rapid Detection

**Figure 5:**
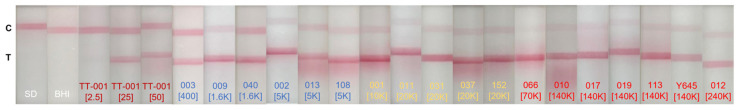
Tetanus Toxin Detection by Immunochromatographic Assay. Immunochromatographic assay results for all study materials arranged by toxin production capacity. Left: Negative controls (SD, sample diluent; BHI, BHI medium) and TT-001 reference standards (2.5, 25, and 50 ng/mL). Right: All 18 *C. tetani* isolates arranged in ascending order of MLD values [400] to [240K] (K = ×1000). Color coding indicates production levels: blue (low), yellow (moderate), red (high producers). All isolates tested positive within 15 min at room temperature, with test line intensities correlating with toxin production levels. T = test line; C = control line.

All TT-001 standards yielded positive results at the tested concentrations (2.5, 25, and 50 ng/mL), and all 18 isolates (10 environmental + 8 clinical) showed positive test results within 15 min (Figure 5). Test line intensities showed general correspondence with toxin production levels, with higher-producing strains typically displaying stronger signals. The negative controls showed no nonspecific reactions, and the control lines developed normally in all measurements.

## 3. Discussion

The ELISA-based method developed in this study demonstrated a strong correlation between measured tetanus toxin antigen levels and mouse bioactivity assays. However, it is important to acknowledge the fundamental distinction between immunoreactive protein quantification and functional neurotoxic bioactivity. Our ELISA measures total tetanus toxin protein, while mouse LD_50_ assays evaluate functional neurotoxic activity in vivo. The observed 0.71–6.03-fold variation between ELISA-derived values and mouse LD_50_ reflects this inherent biological difference, where immunologically detectable but biologically inactive toxin molecules can result from protein degradation, conformational changes, or aggregation during culture storage. Despite this inherent limitation, correlation analysis between ELISA relative potency values and observed MLD values for all 18 isolates revealed a robust positive correlation (r = 0.974, *p* < 0.01, R^2^ = 0.948), demonstrating that ELISA measurements can serve as reliable predictors of relative bioactivity trends across different isolate sources. This addresses a significant gap in the literature, as studies analyzing direct correlations between tetanus toxin antigen levels and biological activity are limited [16]. The ELISA achieved high sensitivity with a lower limit of quantification of 2.4 ng/mL (equivalent to 85.4 LD_50_/mL), which compares favorably with previously reported methods for detecting tetanus toxin.

The choice of polyclonal capture antibodies was based on several considerations: recognition of multiple epitopes on tetanus toxin for improved sensitivity, robust recognition across *C. tetani* strain variations given the observed genetic diversity [9], and cost-effectiveness for assay development. While monoclonal antibodies could theoretically provide improved specificity, our polyclonal system demonstrated excellent specificity with <1% cross-reactivity to other Clostridium toxins, meeting analytical requirements for tetanus toxin detection.

Compared to existing direct detection systems, this ELISA demonstrated superior sensitivity, with approximately 4.1-fold higher sensitivity than Fe-MOF biosensor systems (9.4 ng/mL) [22]. Recent advances in tetanus toxoid potency testing have also explored ELISA-based alternatives to animal bioassays [20], though these methods focus primarily on vaccine standardization rather than direct toxin detection from clinical and environmental samples. While direct comparison with SPR biosensors is challenging due to different reporting units, our method achieved competitive sensitivity among direct toxin detection systems [23].

This represents one of the most sensitive ELISA-based methods reported for detection of tetanus toxin and establishes a new benchmark for immunoassay-based toxin evaluation. Similar advances in bacterial neurotoxin detection have been achieved with botulinum toxin ELISA systems, where electrochemiluminescent methods achieved detection limits of 3–13 pg/mL, demonstrating their potential as ultrasensitive immunoassay approaches [24]. The measurement range (2.4–45.6 ng/mL) effectively covered the diverse toxin-production capabilities observed in both environmental and clinical isolates. The ELISA method successfully assessed isolates with relative potency values and captured the wide range of toxin-production capabilities observed among both environmental and clinical isolates, demonstrating its robustness across diverse analytical applications. The method proved capable of accurately assessing isolates spanning nearly three orders of magnitude in biological activity, validating its utility for both environmental monitoring and clinical diagnosis without extensive animal testing.

In addition to ELISA, we developed an IC assay using the same antibody pair to enable rapid qualitative screening, and successfully detected all 18 isolates within 15 min. The IC assay offers significant advantages for on-site testing and rapid clinical decision-making, enabling immediate qualitative assessment without the need for laboratory equipment or technical expertise [25,26]. Notably, in samples that exhibited high-intensity positive signals, we observed slight variations in the control line intensity, likely due to consumption of the detection antibody (TH-11) at the test line, which is characteristic of sandwich IC systems when dealing with high-concentration analytes [25,26]. This phenomenon did not affect the qualitative interpretation but highlighted the importance of appropriate sample dilution to obtain consistent results.

These two immunoassay formats serve complementary roles, with ELISA being appropriate for precise quantification and IC assays being useful for rapid screening. The ELISA and IC combination provides complementary analytical capabilities that address different operational needs. The IC assay serves as a rapid screening tool for immediate risk assessment in field or emergency settings, while quantitative ELISA provides precise measurements necessary for detailed risk assessment, treatment planning, and epidemiological investigations. This tiered approach optimizes workflow efficiency by reserving time-intensive quantitative analysis for confirmed positive samples, reducing overall analytical burden while maintaining diagnostic accuracy. The combined ELISA and IC approach established in this study provides a comprehensive solution for evaluating tetanus toxin. This combination enables immediate risk assessment, followed by precise quantification when required, significantly enhancing the practical utility of 3R-compliant tetanus toxin evaluation.

Two analytical approaches were validated for the comprehensive evaluation of toxins across all 18 isolates. The ELISA method demonstrated excellent performance across the extended dataset, with both environmental and clinical isolates showing consistent dose–response relationships. The exceptional correlation between different ELISA quantification approaches confirmed their consistency and validity across diverse isolate sources. The method successfully captured the wide range of toxin-production capabilities, spanning nearly three orders of magnitude in biological activity.

The observed differences between ELISA measurements and mouse bioassays reflect fundamental methodological distinctions rather than experimental errors. Analysis of the three representative strains with precise LD_50_ measurements showed that ELISA values ranged from 0.71- to 6.03-fold compared to mouse LD_50_ values, with higher discrepancies observed in low-toxin-producing strains. Mouse bioassays measure actual biological toxicity through in vivo responses, while ELISA quantifies total tetanus toxin protein concentration, including potentially inactive molecules resulting from protein degradation, conformational changes, or aggregation during culture storage. For instance, the low-producer strain KHSU-144303-003 showed a 6.03-fold difference, suggesting significant amounts of immunologically detectable but biologically inactive toxins.

The molecular basis for these discrepancies involves several distinct biochemical pathways that can render tetanus toxin biologically inactive while preserving immunoreactivity. Proteolytic degradation represents a primary mechanism, where environmental or endogenous proteases cleave specific peptide bonds within the toxin molecule. The tetanus toxin structure consists of a heavy chain (100 kDa) responsible for receptor binding and membrane translocation, and a light chain (50 kDa) containing the zinc-dependent catalytic domain, connected by an interchain disulfide bond. Proteolytic cleavage can occur at multiple sites, particularly affecting the light chain’s catalytic activity or disrupting the interchain connection, while major immunogenic epitopes on both chains may remain intact and accessible to antibodies.

Oxidative modifications represent another critical pathway for activity loss. The cata-lytic domain contains a zinc-binding motif (HEXXH) that is particularly susceptible to oxidative damage from reactive oxygen species or metal ion interactions. Oxidation of critical histidine or cysteine residues can abolish the zinc-dependent proteolytic activity essential for neurotoxicity, while the overall protein structure remains sufficiently intact for antibody recognition. Additionally, methionine and tryptophan residues involved in receptor binding may undergo oxidative modifications that eliminate cellular uptake capacity without affecting immunological detection.

Conformational alterations due to environmental stress represent a third major mechanism. Temperature fluctuations, pH changes, ionic strength variations, or dehydration-rehydration cycles during culture storage can cause partial protein unfolding or misfolding. These conformational changes may eliminate the precise three-dimensional structure required for receptor binding or membrane translocation while preserving linear epitopes and secondary structure elements recognized by poly-clonal antibodies. The differential sensitivity of biological function versus immunoreactivity to conformational perturbations explains why some strains show higher ELI-SA-bioactivity discrepancy ratios.

Protein aggregation through intermolecular disulfide formation, hydrophobic interactions, or non-covalent association can render individual toxin molecules biologically inaccessible while maintaining epitope presentation. Aggregated proteins may retain immunoreactivity through surface-exposed epitopes but lose biological activity due to steric hindrance preventing proper receptor interaction or cellular uptake.

The strain-specific variations in ELISA-bioactivity ratios (0.71–6.03-fold) likely reflect differences in intrinsic toxin stability, endogenous protease expression, and culture condition sensitivity among different *C. tetani* lineages. Higher discrepancies in low-producing strains may indicate either inherently less stable toxin variants or suboptimal production conditions leading to increased inactive molecule accumulation.

However, the strong correlation (r = 0.974 for combined MLD prediction across 18 isolates) validated the biological relevance of ELISA measurements as reliable predictors of relative toxin bioactivity across both environmental and clinical specimens. Notably, when the established ELISA-to-bioactivity relationship (0.71–6.03-fold range) was applied to predict the biological activity of the additional 8 clinical isolates, subsequent MLD evaluation confirmed that all isolates fell within the expected range, further validating the predictive utility of the ELISA method for diverse *C. tetani* strains. The ELISA method successfully captured the wide range of toxin-production capabilities observed among both environmental and clinical isolates, providing a robust approach for diverse analytical needs in both environmental monitoring and clinical diagnosis.

The validated method offers significant advantages over conventional mouse bioassays, including reduced measurement time (from days to hours), the elimination of animal use, and the capacity for simultaneous analysis of multiple samples. Matrix interference evaluation confirmed its direct applicability to culture supernatants, supporting its clinical implementation for rapid toxin assessment and decision support in clinical settings. The strong correlation observed in this study demonstrates the practical utility of this method as a reliable 3R-compliant alternative to animal experimentation, representing an essential advancement in tetanus toxin evaluation [27].

Consistent with our previous phylogenetic analysis [9], both environmental and clinical isolates demonstrated remarkable genetic diversity and differences in toxin-production capabilities. This ELISA method proved capable of accurately assessing these characteristics across different specimen types without the need for extensive animal experimentation. This capability is vital for both clinical diagnosis and public health applications, bridging environmental monitoring and clinical practice.

Rapid toxin assessment of both environmental and clinical isolates improves treatment strategies and prognosis determination [6]. The validation with clinical specimens demonstrates the method’s direct applicability in healthcare settings. Notably, in emergencies during disasters and agricultural accidents where the risk of tetanus exposure is elevated [7,8], the combination of 15 min IC screening and precise ELISA quantification significantly enhances patient management and environmental risk assessment across diverse specimen types. While the IC assay enables rapid detection within 15 min, practical implementation for environmental samples requires consideration of sample preparation requirements. Environmental samples containing *C. tetani* require prior cultivation and processing to achieve detectable toxin concentrations, which limits the “on-site” application to situations where basic laboratory facilities are available. The primary advantage of the IC format lies in its ability to provide immediate qualitative results once culture supernatants are prepared, eliminating the need for complex instrumentation and technical expertise typically required for laboratory-based quantification.

This method represents a significant advancement in the implementation of the 3R principle for microbial toxin evaluation [3,27]. The practical implementation pathway is well supported by eliminating animal experimentation while maintaining analytical accuracy and throughput [28], with precedents of regulatory acceptance demonstrated by recent developments in tetanus toxoid potency testing [20]. Although implementation requires the establishment of reference materials and quality control systems, the development of a comprehensive tetanus toxin evaluation system is anticipated through geographical expansion, clinical specimen compatibility, and integration with automation.

This study validated both ELISA and IC methods for measuring tetanus toxin levels across environmental and clinical isolates, demonstrating their validity as alternatives to animal testing. The combined system enables rapid qualitative screening (15 min) and accurate quantitative evaluation across a measurement range of 2.4–45.6 ng/mL (85.4–1805 LD_50_/mL), effectively covering a 258-fold range in relative potency and 600-fold range in biological activity. This establishes a comprehensive alternative methodology that is superior to conventional bioassays and contributes significantly to the implementation of the 3R principles in both environmental monitoring and clinical applications.

Several limitations should be considered when interpreting these results. The bacterial strains used in this study originated from Kumamoto Prefecture, Japan, which represents a geographically confined sampling area. However, our previous comprehensive analysis of 151 *C. tetani* isolates from this region revealed remarkable genetic diversity spanning all major global phylogenetic clades, with multiple lineages coexisting even at individual sampling sites [9]. The 18 strains selected for this study were chosen to represent this phylogenetic diversity across different toxin-production capabilities. While broader geographic validation would strengthen global applicability, the genetic diversity captured in this study spans the major *C. tetani* lineages identified worldwide. Although 18 strains provided proof-of-concept validation, larger multi-center studies would enhance statistical power and generalizability.

## 4. Conclusions

This study successfully developed and validated the comprehensive ELISA and IC assay system as a reliable alternative to animal experimentation for tetanus toxin evaluation. The ELISA demonstrated exceptional analytical performance with a lower limit of quantification of 2.4 ng/mL, excellent linearity (R^2^ = 0.999), and high specificity (<1% cross-reactivity). Strong correlations between ELISA measurements and mouse bioactivity across 18 diverse *C. tetani* isolates (r = 0.974, *p* < 0.01) validated the biological relevance of this approach. The complementary IC assay enabled rapid qualitative screening within 15 min, making the system suitable for both laboratory quantification and field applications.

This approach effectively addressed the wide range of toxin-production capabilities observed in environmental and clinical isolates, spanning nearly three orders of magnitude in biological activity. ELISA measurements showed 0.71–6.03-fold variation compared to mouse LD_50_ values, with predictable patterns that can guide interpretation. This methodology eliminates the need for animal experimentation while reducing evaluation time from days to hours and enabling simultaneous analysis of multiple samples.

The validated system represents a significant advancement in implementing 3R principles for microbial toxin evaluation, with direct applications in clinical diagnosis, environmental monitoring. This breakthrough establishes a new standard for animal-free tetanus toxin assessment and provides a framework for developing similar alternatives for other bacterial toxins.

## 5. Materials and Methods

### 5.1. Tetanus Toxin and Bacterial Strains

TT-001 at a protein concentration of 1.825 mg/mL and tetanus toxoid at a protein concentration of 2.04 mg/mL were both obtained from KM Biologics Co., Ltd., Kumamoto, Japan. TT-001 served as the reference standard for potency determination and analytical method validation, while the toxoid was used for rabbit immunization and preparation of the affinity purification column. Both preparations were stored at −80 °C until analysis.

Overall, 18 representative *C. tetani* strains with diverse toxin-production capabilities were selected for this study. Selection was based on previous phylogenetic analyses and clinical specimen collections [9]. This study included 10 initial isolates (9 environmental + 1 clinical) from previous phylogenetic studies and 8 additional clinical isolates to validate clinical applicability. All strains were cultured in brain heart infusion (BHI) medium at 37 °C for 96 h, followed by centrifugation at 1100× *g* for 20 min. The resulting supernatants were filtered through a 0.2 µm membrane filter and stored frozen at −80 °C until analysis.

### 5.2. ELISA Development and Validation

#### 5.2.1. Production of Polyclonal Antibodies for ELISA Plate Coating

Anti-tetanus toxin polyclonal antibodies were produced by immunizing Japanese white rabbits (female, approximately 3.0 kg, *n* = 2; ARK Resource, Kumamoto, Japan) with tetanus toxoid. The immunization protocol consisted of four intradermal injections of 0.5 mg tetanus toxoid per injection administered at 2-week intervals in the dorsal region. For primary immunization, tetanus toxoid was mixed with equal volumes of Freund’s complete adjuvant (FCA; BD, Franklin Lakes, NJ, USA). For subsequent booster immunizations, Freund’s incomplete adjuvant (FIA; BD, Franklin Lakes, NJ, USA) was used instead of FCA. Ten days after the final immunization, whole blood was collected to obtain immune serum.

The immune serum (5 mL) was purified by a stepwise procedure. Briefly, the se-rum was diluted five-fold with physiological saline (0.9% NaCl; FUJIFILM Wako Pure Chemical, Osaka, Japan), followed by ammonium sulfate precipitation at 45% saturation. A saturated ammonium sulfate solution was gradually added under gentle stir-ring at 4 °C to achieve a final saturation of 45%, and the mixture was incubated over-night at 4 °C. The precipitate was collected by centrifugation at 10,000× *g* for 20 min at 4 °C, dissolved in 5 mL physiological saline, and extensively dialyzed against physiological saline (500 mL, three changes) at 4 °C using a dialysis membrane (Thermo Fisher Scientific, Waltham, MA, USA) with a molecular weight cutoff of 10 kDa.

For affinity purification, a tetanus toxoid-immobilized column was prepared using the Carboxy Link Immobilization Kit (Thermo Fisher Scientific, Waltham, MA, USA) according to the manufacturer’s instructions. Briefly, 3 mg of tetanus toxoid was im-mobilized on 2 mL of resin. The column was equilibrated with 6 mL of binding/wash buffer (1 M NaCl in PBS, pH 7.4; Santa Cruz Biotechnology, Dallas, TX, USA). The dialyzed sample (2 mL) was added to the column and incubated at room temperature for 1 h. After washing with 12 mL of binding/wash buffer, the bound antibody was eluted with 8 mL of elution buffer (Pierce IgG Elution Buffer; Thermo Fisher Scientific, Waltham, MA, USA). Eluted fractions (1 mL each) were collected into tubes containing 50 μL neutralization buffer (1 M Tris-HCl, pH 9.0; Nacalai Tesque, Kyoto, Japan). Antibody concentrations were determined by absorbance at 280 nm using a BioSpectrometer (Eppendorf, Hamburg, Germany). The purity of the antibodies was assessed by SDS-PAGE analysis. High-purity fractions were pooled and concentrated using Amicon Ultra-15 centrifugal filters (Merck Millipore, Burlington, MA, USA). The purified antibodies showed a characteristic IgG band at approximately 140–150 kDa under non-reducing conditions by SDS-PAGE analysis (Figure S1), confirming successful purification of tetanus toxin-specific antibodies.

#### 5.2.2. Preparation of ELISA Plates

Affinity-purified rabbit polyclonal anti–tetanus toxin antibodies were diluted to 5 µg/mL in 10 mM PBS (pH 7.4; Takara Bio, Shiga, Japan) and 0.1% sodium azide (Nacalai Tesque, Kyoto, Japan). Next, 100 µL of the antibody solution was added to each well of flat-bottom 96-well plates (Nunc Immuno Plates; Thermo Fisher Scientific, Waltham, MA, USA). After incubation at 25 °C for approximately 16 h, the plates were washed three times with 10 mM PBS and blocked with 350 µL of blocking solution containing 1% bovine serum albumin (FUJIFILM Wako Pure Chemical, Osaka, Japan), 5% lactose (Nacalai Tesque, Kyoto, Japan), 5% sucrose (Nacalai Tesque, Kyoto, Japan), 1/2500 Proclin300 (Sigma-Aldrich, St. Louis, MO, USA), and 2% Immunoblock (KAC, Kyoto, Japan) in 10 mM PBS. The plates were then incubated overnight at 4 °C. After removing the blocking solution, the plates were dried in a desiccator until the humidity level was below 35%. They were then vacuum-packed in aluminum laminate bags with desiccant and stored at 4 °C until use.

#### 5.2.3. Preparation of HRP-Labeled Detection Antibodies

The detection antibody was prepared by conjugating horseradish peroxidase (HRP; Sigma-Aldrich, St. Louis, MO, USA) to an anti-tetanus toxin mouse monoclonal antibody, TH-11 (FUJIFILM Wako Pure Chemical, Osaka, Japan), using the periodate oxidation method. Briefly, HRP (3.5 mg, corresponding to ~1000 units) dissolved in 1.0 mL of distilled water was oxidized by the addition of 0.2 mL of 0.1 M sodium periodate (NaIO_4_) for 20 min at room temperature in the dark. The oxidized HRP was subsequently dialyzed overnight at 4 °C against 1 mM sodium acetate buffer (pH 4.4) using a dialysis membrane with a molecular weight cutoff of 10 kDa (Thermo Fisher Scientific, Waltham, MA, USA).

Separately, the TH-11 antibody (5.3 mg/mL; total 8 mg) was dialyzed overnight at 4 °C against 0.01 M sodium carbonate buffer (pH 9.5) using the same molecular weight cutoff membrane. The pH of the oxidized HRP solution was adjusted to pH 9.0–9.5 with 0.2 M sodium carbonate buffer, and the dialyzed TH-11 antibody was slowly added to the HRP solution. The conjugation reaction was carried out at a ratio of 1000 units of HRP per 8 mg of TH-11, and the mixture was gently stirred for 2 h at room temperature in the dark. Following conjugation, the reaction mixture was reduced by the addition of 0.1 mL of 0.1 M sodium borohydride (NaBH_4_; Nacalai Tesque, Kyoto, Japan) and incubated for 2 h at 4 °C. The resulting HRP–antibody conjugate was purified by gel filtration chromatography using a Sephacryl S-200 HR (XK16/60; Cytiva, Tokyo, Japan) column equilibrated with 13.3 mM PBS (pH 7.5) as the elution buffer. Eluted fractions with A_403_/A_280_ ratios ≥ 0.4 were pooled and stored at −80 °C with bovine serum albumin (final concentration 10 mg/mL) as a stabilizer.

#### 5.2.4. Measurement Procedure

For the measurements, samples were prepared by two-fold serial dilution using a sample diluent (1% BSA, 0.05% Tween 20 (FUJIFILM Wako Pure Chemical, Osaka, Japan), 1/2500 Proclin 300, and PBS). When sample concentrations exceeded the calibration range, culture supernatants were serially diluted in sample diluent until concentrations fell within the measurement range. Dilution factors were recorded and applied to final concentration calculations. The HRP-labeled detection antibody was diluted to 200 ng/mL in a dilution buffer containing 1% BSA, 50 µg/mL HAMA blocker (Funakoshi, Tokyo, Japan), 1 mM ZnCl_2_ (Nacalai Tesque, Kyoto, Japan), 1 mM MgCl_2_ (Nacalai Tesque, Kyoto, Japan), 0.005% Bromocresol Purple (BCP; Tokyo Chemical Industry, Tokyo, Japan), 1/2500 Proclin 300, 1% Immunoblock, 0.02% Tween 20, 10% HRP Stabilizing Reagent H100 (Nippon Oil & Fats, Tokyo, Japan), 10 µg/mL TRUBlock ULTRA (Meridian Life Science, Memphis, TN, USA), and 10% IMMUNO SHOT-Platinum (Cosmo Bio, Tokyo, Japan) in 0.1 M Tris-0.15 M NaCl (pH 7.2). After adding 100 μL/well of diluted HRP-labeled detection antibodies and 50 μL/well of sample, the plates were incubated at 37 °C for 2 h, washed five times with TBS-T, and 100 µL of 3,3′,5,5′-tetramethylbenzidine solution (SeraCare Life Sciences, Gaithersburg, MA, USA) was added as a chromogenic substrate. The reaction was stopped after incubation at 37 °C for 30 min with 100 µL/well of 1 M sulfuric acid (Nacalai Tesque, Kyoto, Japan), and absorbance was measured at A_450_ nm/A_620_ nm using a microplate reader (Multiskan FC; Thermo Fisher Scientific, Waltham, MA, USA).

#### 5.2.5. Method Validation

The method was validated in accordance with the ICH Q2 (R1) guidelines to assess linearity, accuracy, precision, specificity, and reproducibility [29].

For linearity assessment, TT-001 was serially diluted in the range of 1.4–45.6 ng/mL, and calibration curves were generated using the 4PL regression model. Six replicate measurements were performed per concentration per day over three independent days (*n* = 6 per day). The daily mean was calculated for each concentration, and the overall mean ± standard deviation was derived from the three daily means (*n* = 3 days) to evaluate inter-day variability.

Intermediate precision was assessed at three concentration levels (nominal: 4.56, 11.4, and 22.8 ng/mL). Each concentration was measured eight times per day over three independent days (*n* = 8 per day; total *n* = 24 measurements per concentration), and precision was expressed as the coefficient of variation (CV, %) calculated from all 24 measurements, reflecting combined intra-day (repeatability) and inter-day variability.

Cross-reactivity was assessed using toxins from other Clostridium species (*C. septicum* Lot.5, *C. novyi* Lot.4, and *C. perfringens* Lot.4; all obtained from National Institute of Infectious Diseases (NIID), Tokyo, Japan). Each heterologous toxin was tested in the same concentration range as the TT-001 calibration curve (*n* = 3), and cross-reactivity was calculated as the percentage of absorbance at the highest test concentration relative to TT-001 absorbance at the same concentration, with an acceptance criterion of <5%. Matrix interference was evaluated using the culture supernatant of KHSU-154301-001 diluted in both sample diluent and BHI medium, through parallel line assay. The acceptance criterion was set at *p* > 0.05 (*n* = 3), and statistical analysis was performed using unpaired *t*-test to compare the two matrices.

### 5.3. Animal Bioassays

Female slc:ddy mice (4 weeks old, 18–20 g, SLC, Hamamatsu City, Japan) were used for LD_50_ determination and housed in polycarbonate cages with paper bedding under controlled conditions (12 h light-dark cycle, 22 ± 2 °C, 50–60% humidity) with ad libitum access to food and water. The mice were randomly assigned to groups. At study completion, the mice were euthanized by CO_2_ inhalation. All animals met the inclusion criteria, with no exclusions required.

The LD_50_ of TT-001 was determined using Tetanus Test Toxin Lot 5 (4.4 × 10^6^ LD_50_/mL; NIID, Japan) as the reference standard. Each dilution was administered subcutaneously at a volume of 0.5 mL to four mice per group, and mortality was observed for five days to calculate the LD_50_. For environmental isolates, a preliminary MLD evaluation was performed to establish the relationship between values obtained using ELISA and biological activity and to select representative strains for detailed LD_50_ analysis. Three representative strains with high, moderate, and low toxin-production levels (KHSU-134328-113, KHSU-144313-037, and KHSU-144303-003, respectively) were selected for LD_50_ measurements using the same method.

All animal experiments were approved by the Animal Experimentation Ethics Committee of ARK Resource Co., Ltd. and Kumamoto Health Science University (approval number: AW-21022, 23-08) and were conducted in accordance with the institutional guidelines and 3R principles [14].

### 5.4. Quantitative Analysis Methods

A total of 10 initial isolates (9 environmental + 1 clinical) were initially screened using a validated ELISA with TT-001 as the reference standard. Based on a preliminary evaluation, KHSU-154301-001 was selected as an intermediate-level producer for further analysis and used as a reference standard to predict the MLD values of the remaining nine isolates through ELISA measurements. Subsequently, 8 additional clinical isolates were evaluated using the same methodology to extend clinical validation and demonstrate broader clinical applicability.

Two approaches were employed for the precise quantification of toxins in the 10 initial isolates. First, all isolates were evaluated using the validated ELISA with the 4PL method, with concentrations calculated from the LD_50_ value of TT-001. TT-001 stock solution (6.5 × 10^7^ LD_50_/mL) was appropriately diluted to a working concentration of 1406 LD_50_/mL, which served as the reference standard for both quantification methods. Second, for relative potency determination, TT-001 and initial isolates were serially diluted to generate calibration curves spanning 88.2–1410 LD_50_/mL equivalent concentrations, and a parallel line assay was performed.

### 5.5. Immunochromatographic Assay

The IC assay kit for the rapid detection of tetanus toxin was developed using the same antibody pair used in the ELISA system. The test strips consisted of the following components: (1) sample pad (Millipore, MA, USA), (2) conjugate pad (Millipore, Burlington, MA, USA) containing colloidal gold-labeled TH-11 detection antibody (40 nm gold particles; OD = 1.5, 23.3 μL per strip; prepared by ARK Resource, Tokyo, Japan), (3) nitrocellulose membrane (12 μm pore size; Cytiva, Marlborough, MA, USA) with immobilized capture antibody, (4) absorbent pad (Millipore, Burlington, MA, USA), and (5) backing plastic sheet (Lohmann, Cuxhaven, NRW, Germany).

The test line was prepared by dispensing anti-tetanus toxin polyclonal antibody at 1 mg/mL concentration (1 μL/cm) using an automated dispenser. The control line contained anti-mouse IgG rabbit polyclonal antibody (Proteintech, Rosemont, IL, USA) at 0.2 mg/mL concentration (1 μL/cm). Culture supernatants of all 18 isolates, TT-001 standards (2.5, 25, and 50 ng/mL), and negative controls were tested using 100 μL of undiluted sample per measurement. The results were interpreted as positive or negative after 15 min, based on the visibility of the test line.

### 5.6. Statistical Analysis

Matrix interference was evaluated using an unpaired *t*-test with a significance level of *p* > 0.05. LD_50_ values were calculated using the probit method. A parallel line assay was performed using Bioassay Assist statistical analysis software (Version 3.0, NIID). Calibration curves were fitted using the 4PL method, which is extensively validated for immunoassay data analysis with sigmoidal dose–response relationships [30]. Correlations between the ELISA measurements and mouse bioassays were evaluated using Pearson’s correlation coefficient. Statistical significance was set at *p* < 0.01. Linear regression analysis was performed on the log-transformed data, and the coefficient of determination (R^2^) was calculated.

## Figures and Tables

**Table 1:** ELISA Validation Parameters and Results.

Parameter	TT-001	Acceptance Criteria
Analytical range (ng/mL)	2.4–45.6	Set range
LOQ (ng/mL)	2.4	≤2.4
Linearity (R^2^)	0.999	>0.98
Precision (CV%)	1.7–8.2	<20%
Accuracy	within ±15%	±20%
Repeatability (CV%)	3.3–3.8	<10%
Specificity	<1% cross-reactivity	<5%

**Table 2:** LD_50_ Measurement Results of Representative Strains.

Strain	Toxin Level	Observed LD_50_ (LD_50_/mL)
KHSU134328-113	High producer	1.92 × 10^5^
KHSU144313-037	Moderate producer	2.18 × 10^4^
KHSU-144303-003	Low producer	3.73 × 10^2^

**Table 3:** LD_50_ Determination of TT-001 and Mortality Rates.

LD_50_/mL *	Dose per Mouse (LD_50_/Head)	Deaths/Total	Mortality Rate (%)
3.92	1.96	4/4	100
2.80	1.40	4/4	100
2.00	1.00	4/4	100
1.43	0.7	3/4	75
1.02	0.5	0/4	0

* Dose calculated as LD_50_/mL × 0.5 mL injection volume.

**Table 4:** Comparison of Parallel Line Assay and Single-Point Method for Environmental Isolates.

Strain	Parallel Line Assay		Single-Point (4PL) Method	
	Relative Potency	LD_50_ (LD_50_/mL)	Absorbance	LD_50_ (LD_50_/mL)
KHSU-144303-003	0.025	2250	0.729	1963
KHSU-234315-040	0.029	2585	0.646	3544
KHSU-154306-013	0.120	10,814	0.515	11,635
KHSU-074300-152	0.422	35,703	1.043	41,869
KHSU-154301-001	0.467	42,102	0.560	50,132
KHSU-144313-037	0.516	46,474	0.590	52,465
KHSU-244326-108	0.772	69,481	0.835	70,137
KHSU-134323-066	1.329	119,365	0.741	127,221
KHSU-144313-037	1.514	136,239	0.856	142,984
KHSU-154301-001	1.538	138,235	0.932	153,219

LD_50_ values calculated using diluted TT-001 (1410 LD_50_/mL) as reference standard, corrected for individual sample dilution factors.

**Table 5:** ELISA Relative Potency and MLD Values for Clinical *Clostridium tetani* Isolates.

Strain	Parallel Line Assay		Single-Point (4PL) Method		MLD Value
	Relative Potency	LD_50_ (LD_50_/mL)	Absorbance	LD_50_ (LD_50_/mL)	
NIID-070700-009	0.35	8345	0.695	8582	1600
NIID-072000-002	0.45	10,634	0.882	10,980	5000
NIID-071300-011	0.68	16,037	0.801	19,889	20,000
NIID-071400-031	1.44	34,039	0.757	37,520	20,000
NIID-07AA00-017	7.46	176,392	0.851	169,351	140,000
OPHLC-2022-Y645	7.59	179,303	1.113	222,597	140,000
NIID-072200-019	8.02	189,497	1.011	201,941	140,000
NIID-070300-012	12.91	304,997	0.707	279,572	240,000

LD_50_ values calculated using diluted TT-001 (1410 LD_50_/mL) as reference standard, corrected for individual sample dilution factors.

## Data Availability

The original contributions presented in this study are included in the article/Supplementary Material. Further inquiries can be directed to the corresponding authors.

## Supplementary Materials

The following supporting information can be downloaded at https://www.mdpi.com/article/10.3390/toxins18030133/s1: Figure S1: SDS-PAGE of Purified Anti-Tetanus Antibodies.
