# Supplementary material for: An ELISA-Based Alternative to Mouse Bioassays for Quantitative Evaluation of Tetanus Toxin
Source: Toxins (Basel). 2026 Mar 9;18(3):133. doi: 10.3390/toxins18030133 (PMC13030088; doi:10.3390/toxins18030133)
Supplement: Supplementary file 1 [file toxins-18-00133-s001.zip › toxins-4154174-SI.pdf]

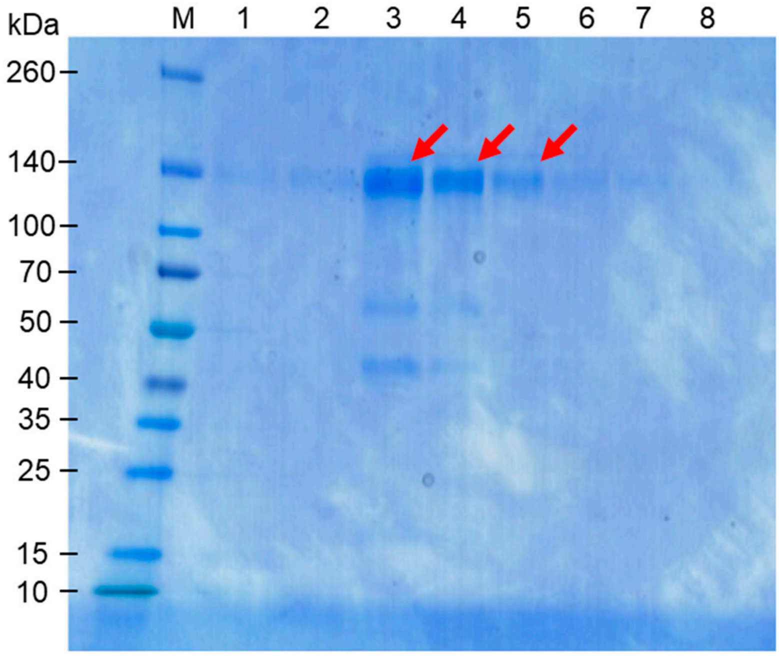

Figure S1. SDS-PAGE of Purified Anti-Tetanus Antibodies

SDS-PAGE of tetanus toxoid affinity-purified rabbit polyclonal antibodies under non-reducing conditions. Lane M, molecular weight marker; lanes 1–8, elution fractions obtained from affinity chromatography. Proteins were visualized by Coomassie Brilliant Blue staining. Major protein bands observed at approximately 140–150 kDa (indicated by red arrows) correspond to intact IgG molecules, indicating successful enrichment of tetanus toxin-specific antibodies. Fractions 3–5, which exhibited the strongest IgG bands, were pooled and used for subsequent ELISA assays.
